# JC polyomavirus (JCV, HPyV2) seropositivity prevalence in healthy subjects: Systematic review and meta-analysis

**DOI:** 10.1371/journal.pone.0341146

**Published:** 2026-01-27

**Authors:** Lenka J. Kimla, David Segal

**Affiliations:** 1 School of Health Sciences, Walden University, Minneapolis, Minnesota, United States of America; 2 School of Health Sciences, Purdue University Global, West Lafayette, Indiana, United States of America; 3 Biomedical Sciences, Keiser University, Orlando, Florida, United States of America; Centers for Disease Control and Prevention, UNITED STATES OF AMERICA

## Abstract

**Background:**

JC polyomavirus (JCV, HPyV2) causes progressive multifocal leukoencephalopathy and has been linked to cancer development. JCV may naturally be highly prevalent in the human population and not just in diseased populations.

**Objective:**

The aim of this study was to the estimate the overall seroprevalence of JCV in the healthy human population by age of subjects, region or country of study, and assay methodology.

**Methods:**

A systematic review and meta-analysis with meta-regression using STATA 18.

**Results:**

Pooled JCV seroprevalence from 25 population-level studies (N = 18,331) was 61% (95% CI, 56% − 66%, *z* = 33.84, *p* < 0.001). Meta-regression and subgroup analyses showed that subject age was the only predictive variable on JCV seroprevalence (*z* = 2.93, *p* = 0.003). Age was not normally distributed across seroprevalence. A detrended normal P-P plot under the cubic model with age on seroprevalence explained 99.9% of variance in the dataset (R^2^ = 0.999). The theories that grounded this study were the ecological systems theory, which support the study result that JCV infection appears ubiquitous in the human population and may be acquired early in life by two years of age.

**Conclusion:**

JCV seroprevalence starts high in early infant age, descends in late childhood/early adulthood, and starts to rise again towards older age, most probably, by viral reactivation due to immune senescence. The overall findings support the hypothesis that JCV is ubiquitous in the healthy human population, and not just diseased populations, which may have implications with JCV treatment, screening, and vaccine development. To the best of our knowledge, this is the first meta-analysis conducted on JCV seropositivity in healthy populations.

## Introduction

JC polyomavirus (JCV, HPyV2) seroprevalence levels in healthy subjects have been reported for select populations and by age from individual studies. These are often small studies conducted in specific age groups that lack infant groups, and which, individually, do not provide the power to estimate JCV seroprevalence in general populations. Meta-analyses on JCV seroprevalence have been conducted in multiple sclerosis (MS), neuromyelitis optica (NMO), and colorectal cancer (CRC) subjects only [1–3]. JCV biology and its association with seroprevalence may be misunderstood, especially in children as JCV seronegativity is frequently equated with lack of JCV infection [4,5].

The virus is thought to be acquired early in childhood via the respiratory and/or fecal-oral routes and transmitted within and outside of families [6]. The archetypal JCV strain results in an infection that is largely asymptomatic and establishes a non-lytic infection in kidney while the prototypical strains are typically found in the bone marrow and brain, formed by rearrangements in its noncoding control region (NCCR) [7]. JCV is neurotropic and lymphotropic. It is thought that the virus crosses the blood-brain barrier via infected B lymphocytes and that it is most probably the archetypal form that is transmissible. A healthy immune system keeps JCV suppressed, however, the virus reactivates under immunosuppression causing life-threatening diseases.

Progressive multifocal leukoencephalopathy (PML), a lytic reactivation of the virus in JCV permissive brain cells, is a threat for patients undergoing immunosuppressive treatments. In JCV nonpermissive cell (not permissive to lytic viral cycle; no VP1 capsid protein made), such as colorectal tissues, JCV is associated with carcinogenesis with the expression of its T-antigen protein [8,9]. The virus appears to exist as one copy per cell [8,10].

Epidemiological studies have used assays to detect JCV antibodies and DNA in serum, urine, or tissues by previously validated methods [11,12]. However, studies conducted with PCR may significantly underestimate JCV DNA presence due to lack of use of topoisomerase I that unwinds the highly supercoiled dsDNA of the virus [13]. Because JCV sheds periodically in urine, serum assays are preferred in detecting past or current JCV infections. Considering JCV’s role in life-threatening diseases such as PML and cancers, more accurate estimates of the risk for JCV reactivation in the general population would be important when treating patients with immunosuppressive treatments. JCV may naturally be highly prevalent in the human population and not just in diseased populations [4]. To-date, no systematic review and meta-analysis has been conducted to assess JCV seroprevalence in healthy (non-diseased) populations, and no study has considered this virus ecologically connected to the human population as part of its environmental ecosystem. Thus, the aim of this quantitative study is to conduct a systematic literature review of JCV seroprevalence studies in healthy populations to estimate its overall seroprevalence by age of subjects, region or country of study, and assay methodology utilizing meta-analyses and meta-regression.

## Methods

This systematic review and meta-analysis were registered on PROSPERO (ID CRD42025632521) and conducted according to the Preferred Reporting Items for Systematic Reviews and Meta-analysis (PRISMA) statement and guidelines [14]. PRISMA checklist was included as S8 Table.

### Literature search strategy

We conducted a systematic literature search to identify publications on JCV seroprevalence in healthy subjects. Peer reviewed publications with full text were selected based on specific inclusion and exclusion criteria [14]. We searched PubMed, CINAHL, and Google Scholar for studies published between January 1, 1971, to December 18, 2024. The following search terms were used: *John Cunningham virus, JCV, JC polyomavirus, JCPyV, seropositivity or antibody or prevalence*, and *population*. A supplemental bibliography review of primary references was conducted using Google Scholar.

### Selection and inclusion criteria

Published studies were eligible for inclusion if they specified JCV cases for healthy subjects from identified populations and reported JCV prevalence in serum by Hemagglutination-inhibition (HI/HAI) test, Immunofluorescence (IF) such as fluorescent bead technology, or ELISA based assays. Studies were excluded if they were detecting JCV deoxyribonucleic acid (DNA) by PCR in tissues or anti-JCV antibody testing in urine.

### Data extraction and quality assessment

Two independent reviewers confirmed the data accuracy and conflicts were resolved by consensus [14]. Extracted data included first authors’ name, the year of publication, JCV seroprevalence, total sample, number of cases, age of subjects, region or country of study, and assay methodology. The quality of each study was assessed by ‘Quality assessment checklist for prevalence studies’ in which a study is rated for a risk of bias by nine items [15].

### Statistical analysis

STATA18 software was used to conduct meta-analysis and meta-regression of single proportion (prevalence) with random effects on JCV seropositivity [16]. The methods included the restricted maximum likelihood (REML) model and Freeman-Tukey transformation (typically used in meta-analyses of single proportion). The significance test of the (pooled) prevalence was calculated by the Z-statistic for the weighted average effect size, with p<0.05 considered to be statistically significant. Heterogeneity was estimated by the Cochrane-Q test and I-squared (I^2^) test statistic.

### Theoretical framework for the study

The theory that grounded this study is the ecological systems theory [17–20]. Widely used in public health, the theory frames the interaction of the human body with the infectious environment throughout the evolution as a living system as well as the human relationship with the physical and social environment [18]. We used the ecological systems thinking to consider that JCV may naturally be highly prevalent in human populations and not just in diseased populations.

## Results

### Literature search

**Fig 1:**
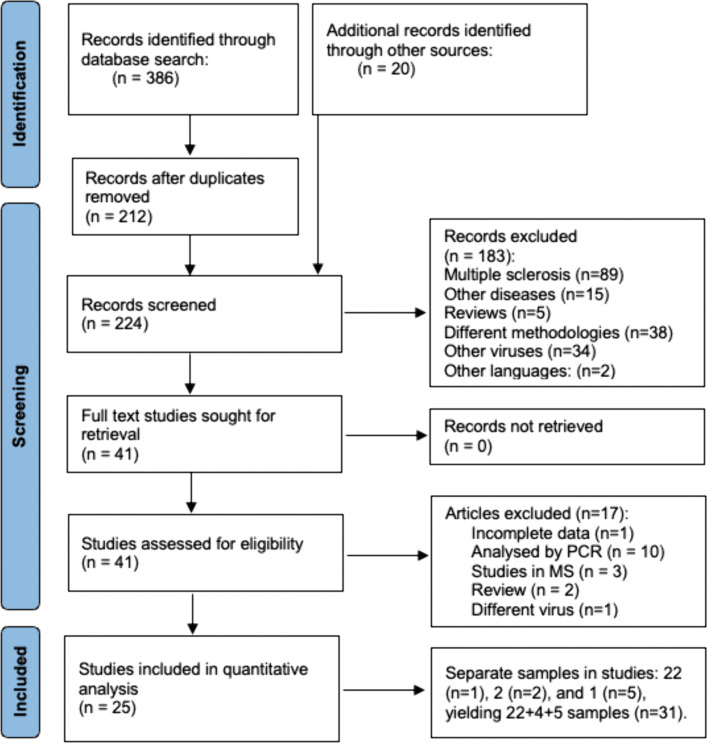
PRISMA flow chart of study selection.

The literature search yielded 386 studies, and after removal of duplicates, 224 articles were screened with 20 additional studies identified through bibliography review (Fig 1). Full texts of 41 publications were assessed, of which 17 were excluded for incomplete data (*n* = 1), analyses by PCR (*n* = 10), diseased populations (*n* = 3), reviews (*n* = 2), or different viruses being studied (*n* = 1). Finally, 25 studies with 31 samples were included in the pooled meta-analysis.

### Characteristics of studies included

Characteristics of the 25 studies published between 1973–2023 with a total population of 18,331 cases are summarized in Table 1. Two studies contained data with two separate samples, and one study had data from five separate databases, thus yielding 31 separate samples (Fig 1). The studies were conducted in Europe (*n* = 17), the United States (*n* = 8), and in APAC (*n* = 6). The methodologies included multiplex fluorescent bead-based serology assays (*n* = 11), HI assay (*n* = 7), ELISA (*n* =7), and GST- or VLP-based ELISA (*n* = 6) (Table 1). Studies reported either mixed aged population or specific age subgroups. For the pooled meta-analysis, the dataset with 31 samples included all subject ages and the variables assay method and country (S1 Table). The additional variable, subject age, was used for subgroup meta-analyses in a dataset with 56 samples and 17,309 cases (S2 and S3 Tables).

**Table 1:** Summary of the 25 studies included in the meta-analysis for pooled JCV seroprevalence.

Study	Region	Method	Age group/s	JCV+	n	Pooled P %
Padget & Walker (1973) [21]	USA	HI	0-50+	324	472	68.8
Gibson et al. (1981) [22]	UK	HI	14-50	208	430	48.4
Taguchi et al. (1982) [23]	Japan	HI	0-50+	96	136	70.5
Coleman et al. (1983) [24]	UK	HI	14-50	34	71	47.9
Chang et al. (2002) [25]	Taiwan	HI	0-50+	646	1022	63.2
Knowles et al. (2003) [26]	UK	HI	0-50+	909	2435	37.3
Rollison et al. (2003) [27]	USA	ELISA	14-50	102	132	77.0
Stolt et al. (2003) [28]	Finland	ELISA	0-50	199	438	45.4
Rollison et al. (2006) [29]	USA	ELISA	14-50	208	276	75.4
Lundstig et al. (2007) [30]	Norway	ELISA	0-50+	284	386	73.6
Egli et al. (2009) [31]	Switzerland	ELISA	14-50+	231	400	57.8
Kean et al. (2009) [32]	USA	GST ELISA	0-50+	691	2030	34.0
Rollison et al. (2009) [33]	USA	ELISA	14-50+	818	1077	76.0
Antonsson et al. (2010) [34]	Australia	Multiplex	14-50+	288	458	62.9
Viscidi et al. (2011) [35]	Italy	VLP ELISA	0-50+	621	945	65.7
Sroller et al. (2014) [36]	Czech Rep	VLP ELISA	0-50+	561	991	56.6
Teras et al. (2015) [37]	USA	Multiplex	50+	336	557	60.3
Gossai et al. (2016)1 [38]	USA	Multiplex	14-50+	256	460	55.6
Gossai et al. (2016)2 [39]	USA	Multiplex	14-50+	177	229	77.3
Karachaliou et al. (2016)1 [40]	Greece	Multiplex	0-50	345	707	48.8
Karachaliou et al. (2016)2 [40]	Greece	Multiplex	0-14	235	690	34.1
Malhotra et al. (2016)1 [41]	EU	Multiplex	50+	35	64	54.7
Malhotra et al. (2016)2 [41]	EU	Multiplex	14-50	77	124	62.1
Malhotra et al. (2016)3 [41]	Asia	Multiplex	50+	151	206	73.3
Malhotra et al. (2016)4 [41]	Asia	Multiplex	50+	86	114	75.4
Malhotra et al. (2016)5 [41]	China	Multiplex	50+	170	209	81.3
Elia et al. (2017) [5]	Italy	GST ELISA	0-14	704	981	71.8
Kamminga et al. (2018) [42]	Netherlands	GST ELISA	14-50+	660	1044	63.2
Bononi et al. (2018)1 [43]	Italy	ELISA	0-50+	151	355	43.0
Bononi et al. (2018)2 [43]	Italy	HI	50+	45	89	51.0
Laine et al. (2023) [44]	Finland	GST ELISA	14-50	318	459	69.3
Total population					18,331	

*Note.* P = prevalence; ELISA = indirect enzyme-linked immunosorbent assay; HI = hemagglutination inhibition test; Multiplex = Fluorescent bead-based multiplex serology; GST ELISA = using GST-VP1 fusion protein; VLP ELISA = using JCV virus-like-particles (VLP)-based ELISA.

### Quality of studies and risk of bias

There was no risk of bias assessment in the 25 studies included in the meta-analysis with the majority as low risk (*n* = 13) and moderate risk of bias (*n* = 12) (S4 and S5 Tables). Most risk items were in the category of generalizability of population to the greater population as most studies focused on specific age categories only, nonrandom selection of population, nondisclosure of randomization, nondisclosure of survey return rate.

### Pooled meta-analysis

**Fig 2:**
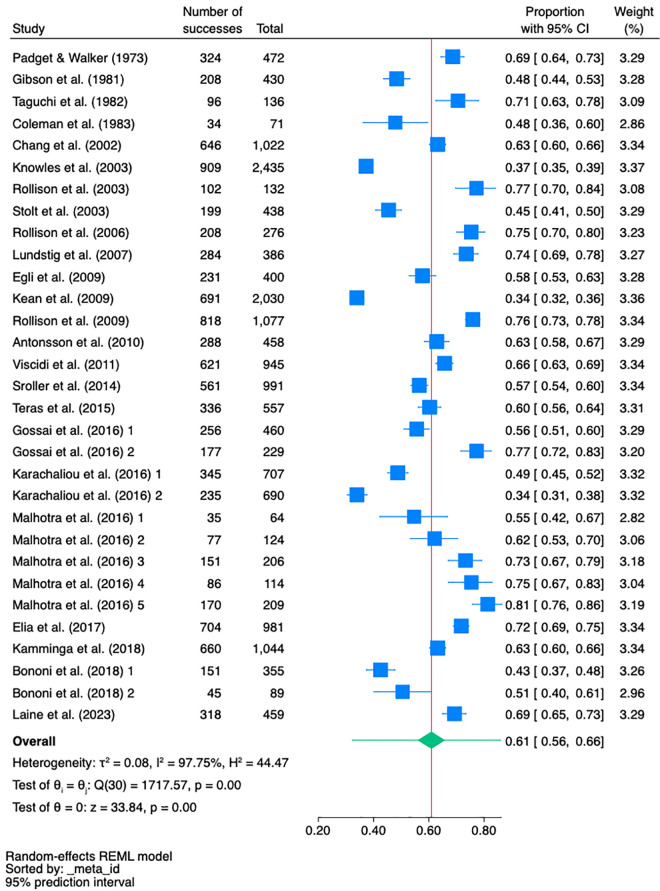
Meta-analysis of pooled JCV seroprevalence in healthy subjects.

In the 25 studies with 31 samples on JCV seroprevalence in healthy subjects (N = 18,331), the mean pooled effect size was 61% (95% CI, 56% − 66%) (Fig 2). The effect size was large and statistically significant (z = 33.84, *p* < 0.001). Heterogeneity was high (I^2^ = 98%) and the χ^2^[Q(30) = 1718, *p* < 0.001] rejected the null hypothesis that the effect size was the same in all the studies. All of the studies are within two standard deviation units of the population (or true effect) indicating that there is no significant inconsistency and no outlier study (S1 Fig).

A meta-regression with random effects revealed that no relationship exists between the seroprevalence and moderators used (method and region) (R^2^ = 0%) (S2 and S4 Figs). The subgroup meta-analyses for region and method show that the subgroup effect sizes did not deviate from the overall pooled effect size (S5 and S6 Figs). S7 Fig shows the funnel plot.

### Subgroup meta-analysis and meta regression by age

**Fig 3:**
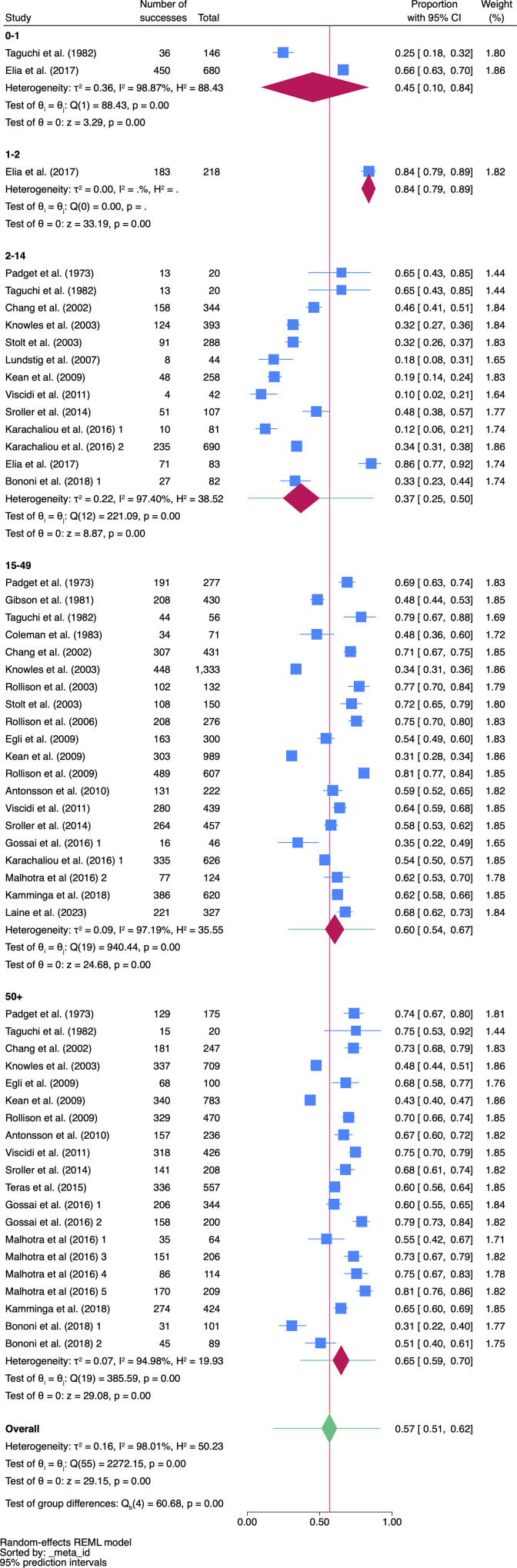
Subgroup meta-analysis of JCV seroprevalence in healthy subjects by age.

The subgroup meta-analysis with the variable ‘age’ revealed an overall mean effect size of 57% (95% CI, 51% − 62%), which was statistically significant (z = 29.15, *p* < 0.001) (Fig 3, S2 and S3 Tables). The chi^2^ test for between group differences [Q(4) = 60.68, *p* < 0.001] indicated moderate effect size differences between groups were present with statistical significance. The chi-squared analysis for differences between studies [Q(55) = 2272, *p* < 0.001] rejected the null hypothesis that the effect size was the same in all the studies and indicating significant heterogeneity among the studies effect sizes. The I^2^ was 98% (Fig 3).

**Fig 4:**
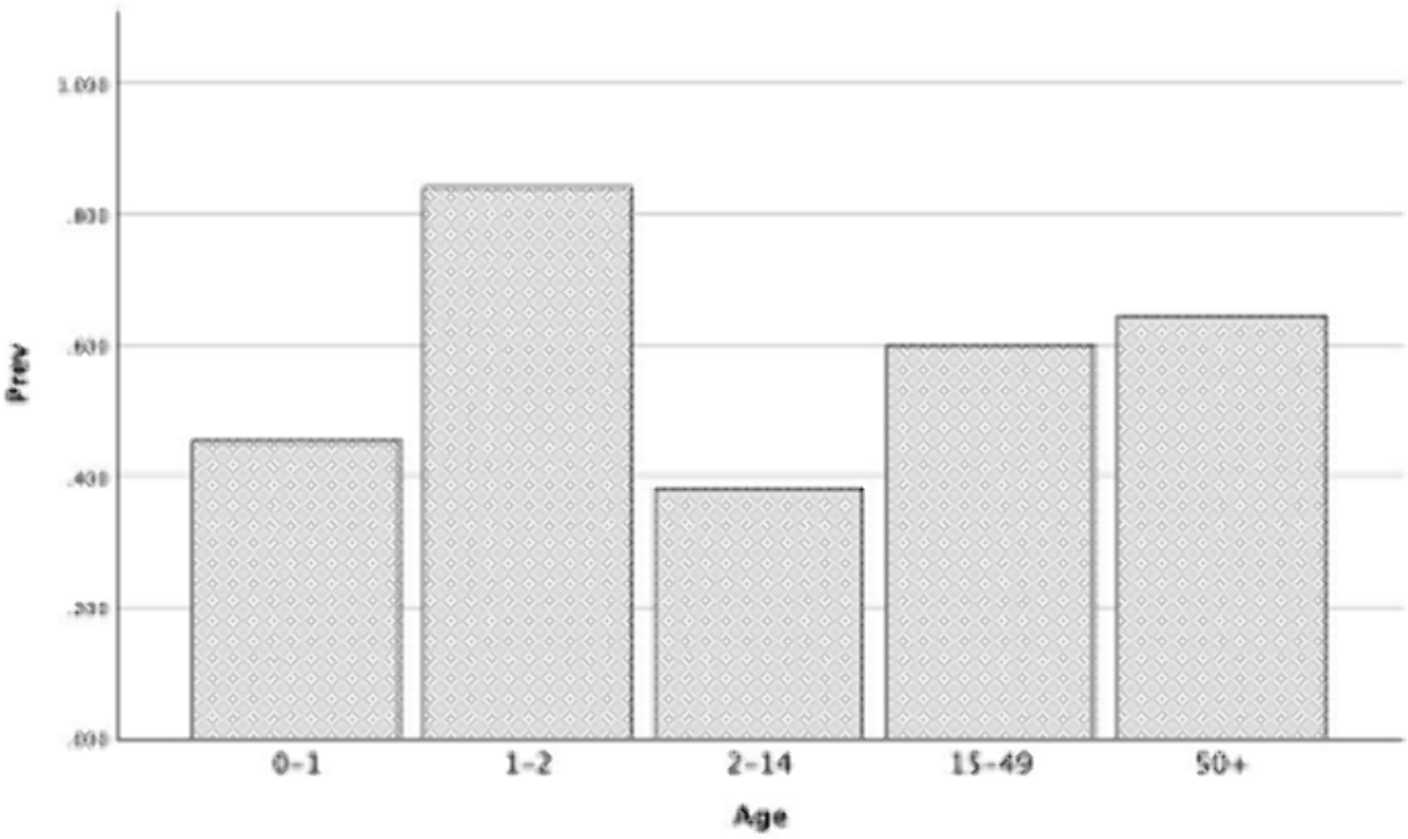
Histogram of JCV seroprevalence in healthy subjects by age.

The overall mean effect size for seroprevalence for each levels of age notably increased from level 1 (children 0–1: 45%, 95% CI: 10% − 84%, z = 3.29, p < 0.001) to level 2 (children 1–2: 84%, 95% CI: 79% − 89%, z = 33.19, p < 0.001) with a subsequent drop in level 3 (children 2–14: 37%, 95% CI: 25% − 50%, z = 8.87, p < 0.001) (Fig 3). The mean seroprevalence continued to increase from level 3 to level 4 (adults 15–49: 60%, 95% CI: 54% − 67%, z = 24.68, p < 0.001) with further increase in level 5 (adult 50 + : 65%, 95% CI 59% − 70%, z = 29.08, p < 0.001). The seroprevalence dynamic for the variable age is shown in Fig 4 displaying a bimodal distribution with a high frequency at level 2 (age 1–2) and level 5 (age 50+).

The results of meta-regression for the subgroup dataset with age as the moderator showed that age accounted for 12.6% of variability in the dataset [R^2^ (%) = 12.64] (S8 Fig). The bubble plot regression line slope shows a significant increase in effect size towards age level 5 (Older 50+) (S9 Fig).

To further examine the effect of the categorical variable age on the continuous variable seroprevalence, while controlling for variables country and assay method, a post hoc partial correlation analysis was conducted in SPSS v.29 with dummy variables for age according to Yang et al. (2017) (S6 Table). The threshold value for Pearson (*r*) is.30 −.49 for moderate effect size. Age older (>50+) had a significant positive and moderate relationship with seroprevalence [*r*(51) =.308, *n* = 56, *p* = .024]. Age children (2−14) had a strong negative significant relationship with seroprevalence [*r*(51) = −.514, *n* = 56, *p* < 0.001]. In the zero-order correlation, region and method had no significant influence in controlling relationship between age and seroprevalence.

**Fig 5:**
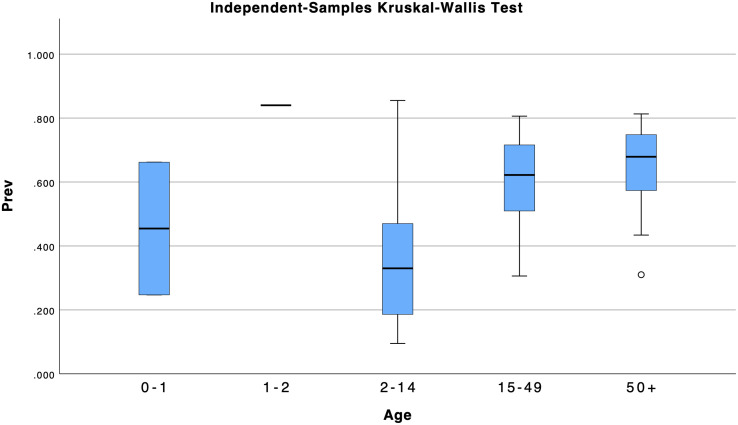
Independent-samples Kruskal-Wallis test box plot of age across prevalence. Prevalence is constant when age = 1-2. It is included in boxplots produced but other output is omitted due to age = 1-2 with only one study included (N = 981).

**Fig 6:**
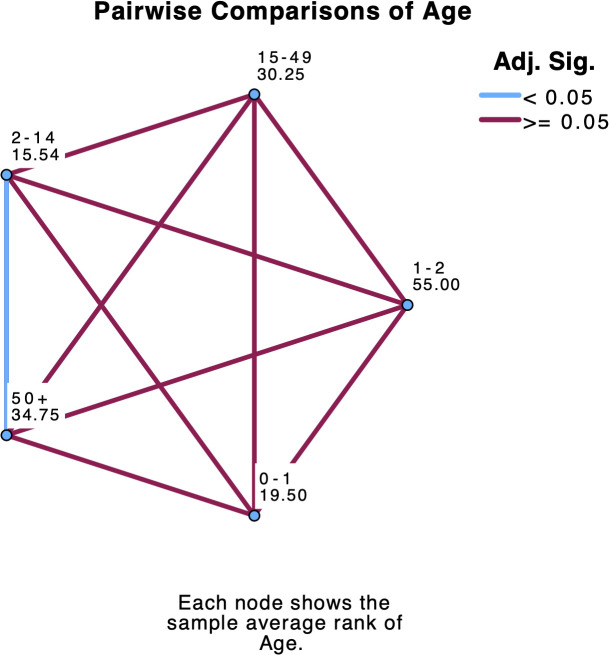
Kruskal-Wallis pairwise comparison of levels of age across prevalence.

A post hoc curve estimation under regression and Shapiro-Wilk test in SPSS v.29 revealed that the relationship between prevalence and age did not fit any linear relationship model and that the distribution of prevalence significantly departed from normality, respectively (S10 Fig, S7 Table). A post hoc non-parametric statistic, the independent-samples Kruskal-Wallis test, revealed significant differences between the medians of the five groups of age on prevalence (H(4) = 14.63, *p* = 0.006) (S7 Table, Fig 5). The pairwise comparisons of age show that only levels of age 2–14 and age 50 + are significantly different (Fig 6).

**Fig 7:**
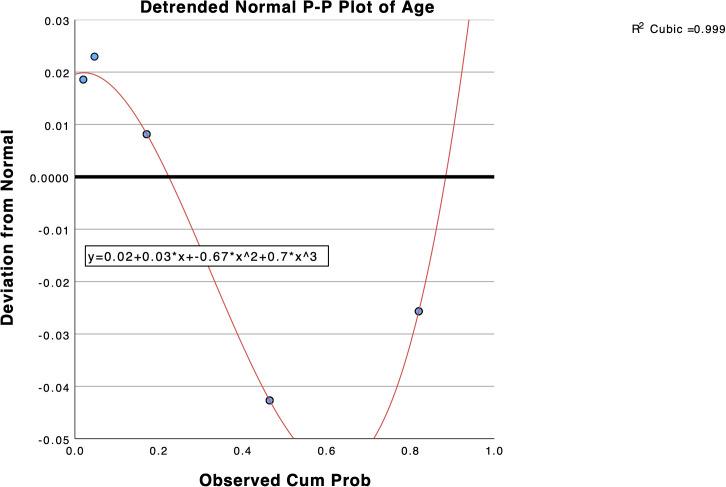
P-P plot curve fit of distribution of age across prevalence (R^2^ = 99.9%).

The post hoc P-P plot statistic revealed that the dataset does not represent a normal distribution with respect to age and prevalence (Fig 7). The detrended normal P-P plot of age against prevalence under the cubic model explained 99.9% of the proportion of variance in the dataset and the R^2^ quantified well for the goodness of fit of the model (R^2^ = 0.999). According to the cubic fitted model, JCV seroprevalence starts high in early infant age, descends in childhood/early adulthood, and then starts to rise in late teens/middle age and towards older age (Figs 3-7).

## Discussion

To the best of our knowledge, this is the first study to conduct a meta-analysis of JCV seroprevalence in healthy populations of subjects. We found that the overall pooled JCV seroprevalence of JCV in healthy populations with 18,331 subjects from the 25 studies was 61% (95% CI, 56% − 66%). Age was the only predictive variable, and it was not normally distributed across JCV seroprevalence. Age exhibited a high JCV seroprevalence in infants and in older individuals (50+), with a decline in late childhood/early adulthood, followed by an increase towards middle and older ages. Viewing the detrended normal P-P plot of age against JCV prevalence under the cubic model explained 99.9% of the variance in the dataset. However, heterogeneity was high (I^2^ = 98%). According to Barker et al. [16], I^2^ has significant limitations in addressing heterogeneity, specifically in prevalence studies. There is no other specific test for assessing heterogeneity in prevalence (proportional) meta-analysis and I^2^ was developed in the context of comparative data. In the context of proportional meta-analysis, heterogeneity is usually high and expected. Studies collecting prevalence and incidence data have differences in time and place where studies were conducted. Also, there were significant differences in age groups collected in each study included, and most omitted collecting infant data, which may be crucial to understanding JCV seroprevalence in healthy populations.

### Seroprevalence predictors

In the individual studies, the overall JCV seroprevalence ranged between 34% to 81% depending on the detection method and region, although neither variable showed a significant effect on JCV seroprevalence, whereas age was a strong predictor.

Our JCV pooled seropositivity in healthy populations of 61% was consistent with JCV seropositivity meta-analysis estimates in patients with multiple sclerosis (MS) (Hanei et al., 2020) [1]. Hanei et al. [1] estimated the JCV pooled seropositivity at 60% in MS patients with varied results from 40% to 80% and significant heterogeneity among studies. It would have been expected that JCV seroprevalence in diseased subjects would have been higher due to JCV reactivation under immunosuppression. However, MS subjects were not subgrouped according to treatment. It is the treatment with natalizumab that is immunosuppressive and triggers JCV reactivation in patients with MS [45]. This indicates a consistency of JCV seroprevalence in the general population and may be reflected of JCV biology with viral latency and reactivation only under an immunosuppressive event [4,46,47].

#### The assay testing method.

This study showed that JCV seropositivity testing method had no effect on prevalence. Taken from all the published evidence, the JCV pooled seroprevalence may not reflect the whole picture of JCV biology in humans and may be an underestimation of the true JCV infection in the healthy human population. It may be important to put JCV seroprevalence in that perspective.

According to Berger et al. [4], a negative JCV antibody status should not be interpreted as absence of JCV infection. In his study, the false negative rate of JCV serology was 37% in those who were JCV positive in urine. Also, JCV seropositive subjects were JCV negative for urine. It showed that seropositivity testing by assays does not identify all patients infected with JCV. The study used ELISA for serum blood testing and JCV DNA presence was tested in urine. The same dynamic was found by Coleman et al. [24] that showed that JCV shedding may increase as immunity decreases in pregnant women.

JCV antibody may not be detectable by antibody assays as JCV may be latent [26]. However, the virus may periodically shed in urine despite a subject’s JCV seronegativity and may not show any pathology. Laine et al. [44] also showed that JCV can seroconvert from positive to negative as a result of seroconversion waning.

In this meta-analysis all assays appeared equal. The seroprevalence of JCV varies widely among different studies which could be explained by differences in sensitivity and specificity for each assay used [26]. Hamilton et al. [11] found that the JCV HI and ELISA assays correlated well with one another and did not cross-react with BK virus with which JCV shares homology. ELISA was developed later than HI, and more recently, tests using recombinant antigens such as VLPs and GST-captured ELISA helped to overcome common limitations in producing viral antigens in cell culture, especially for JCV [26]. Novel assays to measure polyomavirus seroreactivity use different fusion proteins as substrate for the VP1 antigens to detect serum antibodies against JCV and these are produced in different vehicles (bacteria, viruses, yeast) [12].

Confirming JCV infection may depend on what test and tissue is used. PCR has not been a variable studied in this meta-analysis, however, JCV DNA has been found in healthy colorectal tissue with the use of PCR [48]. JCV DNA is highly negatively supercoiled, therefore, researchers need to use topoisomerase I to unwind the dsDNA to anneal the primer successfully [13]. However, since most studies have not used it, JCV DNA findings may be underestimated in studies and erroneously conclude the absence of JCV DNA in tissues [8,13]. With the use of topoisomerase I and PCR, Izi et al. [13] located JCV DNA in 60% of CRC tissues when none was found without the use of topoisomerase I. JCV T-antigen protein expression has only been found in cancer tissues and none in healthy tissues despite JCV DNA presence in both [8]. JCV may be hard to detect with just one test in healthy subjects unless reactivated by an immunosuppressive event. It is an opportunistic human pathogen [49].

#### Subgroup analysis with age.

Subgroup analysis and meta-regression was conducted to explore the heterogeneity with five levels of age (infants 0–1, Infants 1–2, children 2–14, adults 15–49, older 50+). JCV seroprevalence in healthy populations with 17,309 subjects from the 25 studies including 56 samples with the variable subject age was 57% (95% CI, 51% − 62%). Meta-regression showed that the moderator age was the only independent variable that had an effect on the dependent variable prevalence with statistically significant differences between the groups of age (z = 2.93, *p* < 0.003) exhibiting a high seroprevalence in infants and in older ages (50+). This novel finding extends the results of age dependent changes in JCV seroprevalence from previously published studies which did not include infants in their data and analysis. Increasing JCV seroprevalence by age has long been noted in the literature. However, Padget and Walker [21] observed early on that the highest rate of JCV seroconversion occurred before the age of 14, with a slight increase between the ages 15 and 50, and another after the ages of 50. They found the highest HI antibody titers in the sera of a 5-year-old child and 75-year-old woman suggesting that JCV infection occurs early in life [6]. After an initial infection in early childhood, the virus then enters a latent period in the kidneys and only reactivates during immunosuppression, which would explain the rise in seroprevalence later in life due to immune senescence [5,9].

#### JCV seropositivity status in children.

Elia et al. [5] concluded from a longitudinal serological study of 981 infants that JCV primary infection occurs very early in life before the age of six month. JCV seropositivity was significantly lower in children less than six months versus children older than six month (*p* < 0.0001). Knowles [26] noted that overall JCV seroprevalence can vary depending on how many children were included in the sample. Most studies and samples of children in this meta-analysis were small with limited ages, and most excluded infants. The seroprevalence in children 0–14 years of age ranged between 10% to 84% in the 56 samples in this meta-analysis in the 24 studies included, specifically seroprevalence was 45% ages 0–1, 84% ages 1–2, and 37% ages 2–14. This disagrees with the general consensus in literature that children’s seropositivity for JCV is around 33.3% based on evidence at the time, which excluded infants in their data [45]. Elia et al. [5] who studied 981 infants aged 1 day to 3 years found that their JCV seroprevalence increased over time from 46.1% (1-month-old) to 80.7% (12 months old), 85.9% (24-months-old), and 85.5% (36-month-old) (*p* = 0.001). A large percentage of infants become seropositive by age two, which is very important for potential future vaccinations [5]. Elia et al. [5] noted that serological evidence on JCV is rarely studied in early childhood. In later childhood, as the immune system matures along with immunosurveillance for JCV, the virus enters latency with asymptomatic persistence and antibodies may wane. This can affect seroprevalence rates. Hennes et al. [45] conclusion that since children’s JCV seropositivity status is lower than in adults (33% versus 60%), the lower the risk for drug induced PML in children is incorrect. Natalizumab is an immunosuppressive drug used to treat patients with MS and is associated with the presence of anti-JCV antibodies [50]. To-date, Natalizumab has not been approved for the treatment of children with MS as the safety in this population has not been established [51].

#### JCV antibody waning.

Antibody waning was also found in adult populations affecting JCV seropositivity. Laine et al. [44] found in a longitudinal Finnish study that JCV antibodies can wane at about 5% in a sample of pregnant women. However, in a sample of MS patients from a 6-year longitudinal study, JCV antibody status changed from negative to positive and back again in 17% of these patients [50]. This, again, showcases that JCV seronegativity does not always indicate a lack of underlying JCV infection as per Berger et al. [4].

#### JCV seropositivity status in adults.

In the study of 327 pregnant women and 132 of their spouses, JCV seroprevalence varied between 59–68% in women with mean age 25.5 years and 66–72% in their spouses with mean age 29 years [44]. This agrees with the mid to upper range of JCV seropositivity in this meta-analysis for adults aged 15–49 years, which was 30.6–78.6%. If data were available, stratifying age groups into smaller 10-year intervals would be informative. Laine et al. [44] and Knowles [26] also found that JCV seropositivity was more common in men than women. Gender could be another variable that affects JCV seroprevalence, although other studies such as Padget and Walker [21] did not report a difference. SV40 virus, another polyomavirus homologous with JCV, was found to have higher seroprevalence in men than women. This was thought to be due to the fact than men work outside more often than women who may come in contact with the antigen and stimulate an immune response [52]. According to Knowles [26], sporadic JCV reactivation may be a factor as well as an inability to suppress JCV replication in some individuals’ seropositivity. Additionally, it is not known how the presence of IgM antibody relates to viral shedding in healthy adults and how IgM presence relates to age [26]. However, according to Knowles [26], the excretion of stable genomes over time suggests JCV persistence in humans rather than reinfection.

#### JCV seropositivity status in older adults (>50).

While JCV seropositivity trended higher at 65% in older adults aged 50 and higher compared to adults aged 15–49 years of age, it was significantly higher than children aged 2–14 years of age (*p* < 0.001). The range of JCV seroprevalence in older adults was 31% to 81% (Fig 3). These results are consistent with literature. Padget and Walker [21] stated, when the virus was discovered, that the highest JCV titers were in in a 51-year-old man, 75-year-old women, and 90-year-old woman in their sample. Knowles [26] summarized that JCV seroprevalence increases more gradually into old age based on studies available at the time.

### Theoretical framework interpretation of findings

JCV is exclusively a human pathogen [53]. It is of ancient evolutionary origin having evolved from plasmids and co-evolved with humans not causing them harm unless immunosuppression takes place [54]. This is directly related to the ecological model and systems theory, which points out that humans interact with their environment [17,18]. JCV is acquired very early in life through fecal oral route and possibly respiratory route by interacting with human sociocultural environment [5,18,.48]. JCV is spread within families and outside of family groups [26]. Sewage and oyster bed studies indicate that JCV is present via asymptomatic shedding of JCV virions in urine [6,55–58]. Our study found that JCV seropositivity is high in infants, subsides in late childhood/early adulthood, and subsequently increases with age, most probably by viral reactivation due to immune senescence [4,24,59]. Therefore, the overall findings support the hypothesis that JCV is ubiquitous in the human population. Prevalence of JCV infection in the healthy population may be reflective of patterns of the EBV virus infection, a ubiquitous human pathogen acquired early in life which becomes latent [60].

## Limitations of the study

Considering this meta-analysis analyzed seroprevalence in healthy populations, this study is limited to the data collected from the populations and their underlying characteristics in the sample, which may vary across studies included. The publication bias cannot be assessed in this study of prevalence as the egger’s test and funnel plots were developed in the context of comparative data [16]. The assumption that positive data are published more often is not necessarily true for data of proportions as there is no consensus on what the positive results for proportions (aka prevalence) are. Barker et al. [16] does not recommend that publication bias be used for meta-analyses of proportions. Similarly, I^2^ has its limitation in assessing heterogeneity in prevalence studies as in this context heterogeneity is usually high and expected as discussed [16]. Each study included may have had differences in how its methodology, even if the same was used as other studies, had been carried out or reported detection limits. Differences in specific age groups and sex collected by each study included in the analysis may have affected the prevalence outcome. Not all regions of the world have been represented in this analysis. Some studies had small sample sizes.

Not all studies were of epidemiological study quality, but some were case-control studies and their healthy controls for JCV seroprevalence were included in this analysis. Therefore, some samples were not randomly selected, which was captured in the quality assessment checklist for prevalence studies [15]. Only three independent variables were assessed to influence the outcome even though potential confounding variables may exist which was not available in each study. The number of studies available for the three variables was also limited and the analysis would have benefited from more studies of higher quality using large samples with all age groups represented. Infants were not represented in most studies which may have affected the outcome.

The strength of this study is the large, pooled sample size of 18,331 individuals from 16 countries and two regions (EU and Asia) to assess the pooled JCV seroprevalence in healthy populations, which increased the power of the test versus individual studies with small samples. This study allowed for subgroup analyses by subject age, country, and assay methodology to assess the sources of heterogeneity. Most importantly, this study showcases high seroprevalence in infants and children and corrects the general misconception that JCV seroprevalence is low in children and points out the ubiquitous nature of JCV in human population.

## Recommendations

The limitations described in this meta-analysis lay basis for future studies analysing JCV seroprevalence in healthy populations. Future research should consider the effect of age and have representation of all age groups, including infants, larger sample sizes, and use the latest methodology to assess seropositivity. A study conducting different test to assess JCV infection in healthy subjects such as seropositivity, JCV presence in urine, and PCR test from a tissue, would be informative.

## Implications

Public health programs aim at improving the health of populations and prevent diseases in populations. As more knowledge is gained on the biology of JCV and the prevalence of infection by JCV in healthy populations, not just seropositivity with potentially erroneous conclusions, there may be a benefit in developing a vaccine to prevent its numerous associated diseases and the potential threat an individual might face should they have an immunosuppressive event. Elia et al. [5] demonstrated that most infants are JCV infected by the age of two years. The opportunity to vaccinate would ideally be during the first 6 months of life [5]. Recommendations for practice would be to seek patient’s JCV status not just by seropositivity but also by urine and tissue tests by different methods such as PCR with the use of topoisomerase I.

## Conclusions

To the best of our knowledge, this is the first meta-analysis conducted on JCV seropositivity in healthy populations. The pooled JCV seroprevalence in healthy populations was 61% and dependent on age with a high seroprevalence in infants, a subsequent decline in late childhood/early adulthood followed by an increase in older ages. The findings of this systematic literature review indicate that JCV is ubiquitous in human population with most infections acquired prior to 2 years of age in majority of infants. Researchers and clinicians would be advised not to rely heavily on JCV seropositivity status as the virus can be latent and escape immunosurveillance. Future studies should focus on a panel of JCV tests in serum, urine, and tissue, which would include testing by PCR (with the use of topoisomerase I) and the latest immunoassays to uncover the presence of underlying JCV infection. The study findings provide valuable insight into the impact that JCV infection may have on public health and the benefits of JCV vaccination efforts.

## Supporting information

S1 Table Codebook for pooled meta-analysis variables. (PDF)

S2 Table Codebook for subgroup meta-analysis variables. (PDF)

S3 Table Summary of age subgroups from the 25 studies included in the meta-analysis for subgroup JCV seroprevalence. (PDF)

S4 Table Risk of bias assessment for prevalence studies. (PDF)

S5 Table Quality assessment checklist for prevalence studies. (PDF)

S6 Table Partial correlation between independent variables age and dependent variable prevalence controlling for region and method. (PDF)

S7 Table Independent-samples Kruskal-Wallis test output (SPPS v.29) on prevalence across age. (PDF)

S8 Table PRISMA checklist. (PDF)

S1 Fig Galbraith plot for assessing heterogeneity in the pooled meta-analysis. (PDF)

S2 Fig Meta-regression output from STATA 18 for pooled meta-analysis dataset. (PDF)

S3 Fig Bubble plot with region as moderator. (PDF)

S4 Fig Bubble plot with method as moderator. (PDF)

S5 Fig Subgroup meta-analysis for JCV seroprevalence in healthy subjects by method in the pooled dataset. (PDF)

S6 Fig Subgroup meta-analysis for JCV seroprevalence in healthy subjects by region in the pooled dataset. (PDF)

S7 Fig Funnel plot for pooled meta-analysis of JCV seropositivity for publication bias. (PDF)

S8 Fig Meta-regression for the subgroup age effect on seroprevalence. (PDF)

S9 Fig Bubble plot with age as moderator. (PDF)

S10 Fig Curve estimation of independent categorical variable age on dependent continuous variable prevalence. (PDF)
