# Correction: JC polyomavirus (JCV, HPyV2) seropositivity prevalence in healthy subjects: Systematic review and meta-analysis

**DOI:** 10.1371/journal.pone.0343707

**Published:** 2026-02-23

**Authors:** The PLOS One Staff

One of the corresponding author’s email addresses is incorrect. David Segal’s email address is: david.segal@purdueglobal.edu. The publisher apologizes for the error.
