# Supplementary material for: JC polyomavirus (JCV, HPyV2) seropositivity prevalence in healthy subjects: Systematic review and meta-analysis
Source: PLoS One. 2026 Jan 27;21(1):e0341146. doi: 10.1371/journal.pone.0341146 (PMC12843548; doi:10.1371/journal.pone.0341146)
Supplement: S1 Table — (PDF) [file pone.0341146.s001.pdf]

S1 Table. Codebook for pooled meta-analysis variables.

Variable Numeric Value	Region (Nom)		Method (Nom)	
	Label	Freq (%)	Label	Freq (%)
1	USA	8 (25.8)	HI	7 (22.6)
2	EU	17 (54.8)	ELISA	7 (22.6)
3	AsiaAPAC	6 (19.4)	GSTVLP ELISA	6 (19.4)
4	-		Multiplex	11 (35.4)
Total (N)		31 (100)		31 (100)
