# Supplementary material for: JC polyomavirus (JCV, HPyV2) seropositivity prevalence in healthy subjects: Systematic review and meta-analysis
Source: PLoS One. 2026 Jan 27;21(1):e0341146. doi: 10.1371/journal.pone.0341146 (PMC12843548; doi:10.1371/journal.pone.0341146)
Supplement: S2 Table — (PDF) [file pone.0341146.s002.pdf]

S2 Table. Codebook for subgroup meta-analysis variables.

Var (Type)	Region (Nom)		Method (Nom)		Age (Ord)	
Value	Label	Freq (%)	Label	Freq (%)	Label	Freq (%)
1	USA	14 (25)	HI	16 (28.6)	0 – <1	2 (3.6)
2	EU	30 (53.6)	ELISA	11 (19.6)	1 - 2	1 (1.8)
3	AsiaAPAC	12 (21.4)	GSTVLP	15 (26.8)	2+ -14	13 (23.2)
4	-	-	Multiplex	14 (25)	15-49	20 (35.7)
5	-	-	-		>49	20 (35.7)
Total (N)		56 (100)		56 (100)		56 (100)
