# Supplementary material for: JC polyomavirus (JCV, HPyV2) seropositivity prevalence in healthy subjects: Systematic review and meta-analysis
Source: PLoS One. 2026 Jan 27;21(1):e0341146. doi: 10.1371/journal.pone.0341146 (PMC12843548; doi:10.1371/journal.pone.0341146)
Supplement: S3 Table — (PDF) [file pone.0341146.s003.pdf]

S3 Table. Summary of age subgroups from the 25 studies included in the meta-analysis for subgroup JCV seroprevalence. P = prevalence; Age group 1 = Children 0-<1 years of age; Age group 2 = Children 1-2; Age group 3 = >2 -14; Age group 4 = Adults 15-49 years of age; Age group 5 = Older adults inclusive 50+ years of age.

Study	Age Group	JCV+	n	P %
Padget et al. (1973)	3	13	20	65.0
Padget et al. (1973)	4	191	277	69.0
Padget et al. (1973)	5	129	175	76.4
Gibson et al. (1981)	4	208	430	48.4
Taguchi et al. (1982)	1	36	146	24.7
Taguchi et al. (1982)	4	44	56	78.6
Taguchi et al. (1982)	5	15	20	75.0
Taguchi et al. (1982)	3	13	20	65.0
Coleman et al. (1983)	4	34	71	47.9
Chang et al. (2002)	3	158	344	46.0
Chang et al. (2002)	4	307	431	71.2
Chang et al. (2002)	5	181	247	73.4
Knowles et al. (2003)	3	124	393	31.6
Knowles et al. (2003)	4	448	1333	33.8
Knowles et al. (2003)	5	337	709	47.5
Rollison et al. (2003)	4	102	132	77.0
Stolt et al. (2003)	3	91	288	32.0
Stolt et al. (2003)	4	108	150	72.0
Rollison et al. (2006)	4	208	276	75.4
Lundstig et al. (2007)	3	8	44	18.2
Egli et al. (2009)	4	163	300	54.3
Egli et al. (2009)	5	68	100	68.0

Study	Age Group	JCV+	n	P %
Kean et al. (2009)	3	48	258	18.6
Kean et al. (2009)	4	303	989	30.6
Kean et al. (2009)	5	340	783	43.4
Rollison et al. (2009)	4	489	607	80.6
Rollison et al. (2009)	5	329	470	70.0
Antonsson et al. (2010)	4	131	222	59.0
Antonsson et al. (2010)	5	157	236	66.4
Viscidi et al. (2011)	3	4	42	9.50
Viscidi et al. (2011)	4	280	439	63.8
Viscidi et al. (2011)	5	318	426	74.6
Sroller et al. (2014)	3	51	107	47.0
Sroller et al. (2014)	4	264	457	57.0
Sroller et al. (2014)	5	141	208	67.8
Teras et al. (2015)	5	336	557	60.3
Gossai et al. (2016) 1	4	16	46	34.0
Gossai et al. (2016) 1	5	206	344	60.0
Gossai et al. (2016) 2	5	158	200	79.0
Karachaliou et al. (2016) 1	3	10	81	12.3
Karachaliou et al. (2016) 1	4	335	626	53.5
Karachaliou et al. (2016) 2	3	235	690	34.1
Malhotra et al (2016) 1	5	35	64	54.7
Malhotra et al (2016) 2	4	77	124	62.1
Malhotra et al (2016) 3	5	151	206	73.3
Malhotra et al (2016) 4	5	86	114	75.4
Malhotra et al (2016) 5	5	170	209	81,3
Elia et al. (2017)	1	450	680	66.2

Study	Age Group	JCV+	n	P %
Elia et al. (2017)	2	183	218	84.0
Elia et al. (2017)	3	71	83	85.5
Kamminga et al. (2018)	4	386	620	62.3
Kamminga et al. (2018)	5	274	424	64.6
Bononi et al. (2018) 1	3	27	82	33.0
Bononi et al. (2018) 1	5	31	101	31.0
Bononi et al. (2018) 2	5	45	89	51.0
Laine et al. (2023)	4	221	327	67.8
Total population			17,309	
