# Supplementary material for: JC polyomavirus (JCV, HPyV2) seropositivity prevalence in healthy subjects: Systematic review and meta-analysis
Source: PLoS One. 2026 Jan 27;21(1):e0341146. doi: 10.1371/journal.pone.0341146 (PMC12843548; doi:10.1371/journal.pone.0341146)
Supplement: S4 Table — (PDF) [file pone.0341146.s004.pdf]

S4 Table. Risk of bias assessment for prevalence studies. Source: Quality assessment checklist for prevalence studies adapted from Hoy et al. [15]; 0 score = low risk per bias item; 1 score = high risk per bias item. T = Total score.

1st Author	Q1	Q2	Q3	Q4	Q5	Q6	Q7	Q8	Q9	T	Risk of bias
Padget	0	0	1	1	0	0	0	0	0	2	Low
Gibson	1	0	0	1	0	0	0	0	0	2	Low
Taguchi	1	0	0	0	0	0	0	0	0	1	Low
Coleman	1	1	1	0	0	0	0	0	0	3	Low
Chang	0	0	1	1	0	0	0	0	0	2	Low
Knowles	1	0	0	0	0	0	0	0	0	1	Low
Rollison 1	1	1	1	1	0	0	0	0	0	4	Moderate
Stolt	1	1	0	1	0	0	0	0	0	4	Moderate
Rollison 2	1	1	1	1	0	0	0	0	0	4	Moderate
Lundstig	1	1	1	1	0	0	0	0	0	4	Moderate
Egli	1	1	1	1	0	0	0	0	0	4	Moderate
Kean	1	1	1	1	0	0	0	0	0	4	Moderate
Rollison	1	1	1	1	0	0	0	0	0	4	Moderate
Antonsson	1	0	0	0	0	0	0	0	0	1	Low
Viscidi	1	1	1	1	0	0	0	0	0	4	Moderate
Sroller	0	1	0	1	0	0	0	0	0	2	Low
Teras	1	1	1	1	0	0	0	0	0	4	Moderate
Gossai, 1,2	1	1	1	1	0	0	0	0	0	4	Moderate
Karachaliou	1	0	1	0	0	0	0	0	0	2	Low
Malhotra	1	1	1	1	0	0	0	0	0	4	Moderate
Elia	1	0	1	1	0	0	0	0	0	3	Low
Kamminga	1	0	0	0	0	0	0	0	0	1	Low
Bononi	0	0	0	0	0	0	0	0	0	0	Low

1st Author	Q1	Q2	Q3	Q4	Q5	Q6	Q7	Q8	Q9	T	Risk of bias
Laine	1	0	1	1	0	0	0	0	0	3	Low
