# Supplementary material for: JC polyomavirus (JCV, HPyV2) seropositivity prevalence in healthy subjects: Systematic review and meta-analysis
Source: PLoS One. 2026 Jan 27;21(1):e0341146. doi: 10.1371/journal.pone.0341146 (PMC12843548; doi:10.1371/journal.pone.0341146)
Supplement: S5 Table — (PDF) [file pone.0341146.s005.pdf]

S5 Table. Quality assessment checklist for prevalence studies (adapted from Hoy et al [1])

Name of author(s):

Year of publication:

Study title:

Risk of bias items	Risk of bias levels	Points scored
1. Was the study’s target population a close representation of the national population in relation to relevant variables, e.g. age, sex, occupation?	**Yes** (**LOW RISK**): The study’s target population was a close representation of the national population.	0
	**No** (**HIGH RISK**): The study’s target population was clearly NOT representative of the national population.	1
2. Was the sampling frame a true or close representation of the target population?	**Yes** (**LOW RISK**): The sampling frame was a true or close representation of the target population.	0
	**No** (**HIGH RISK**): The sampling frame was NOT a true or close representation of the target population.	1
3. Was some form of random selection used to select the sample, OR, was a census undertaken?	**Yes** (**LOW RISK**): A census was undertaken, OR, some form of random selection was used to select the sample (e.g. simple random sampling, stratified random sampling, cluster sampling, systematic sampling).	0
	**No** (**HIGH RISK**): A census was NOT undertaken, AND some form of random selection was NOT used to select the sample.	1
4. Was the likelihood of non-response bias minimal?	**Yes** (**LOW RISK**): The response rate for the study was ≥75%, OR, an analysis was performed that showed no significant difference in relevant demographic characteristics between responders and non- responders	0
	**No** (**HIGH RISK**): The response rate was <75%, and if any analysis comparing responders and non-responders was done, it showed a significant difference in relevant demographic characteristics between responders and non-responders	1
5. Were data collected directly from the subjects (as opposed to a proxy)?	**Yes** (**LOW RISK**): All data were collected directly from the subjects.	0
	**No** (**HIGH RISK**): In some instances, data were collected from a proxy.	1
6. Was an acceptable case definition used in the study?	**Yes** (**LOW RISK**): An acceptable case definition was used.<br>**No** (**HIGH RISK**): An acceptable case definition was NOT used	0<br>1
7. Was the study instrument that measured the parameter of interest (e.g. prevalence of low back pain) shown to have reliability and validity (if necessary)?	**Yes** (**LOW RISK**): The study instrument had been shown to have reliability and validity (if this was necessary), e.g. test-re- test, piloting, validation in a previous study, etc.	0
	**No** (**HIGH RISK**): The study instrument had NOT been shown to have reliability or validity (if this was necessary).	1
8. Was the same mode of data collection used for all subjects?	**Yes** (**LOW RISK**): The same mode of data collection was used for all subjects.	0
	**No** (**HIGH RISK**): The same mode of data collection was NOT used for all subjects.	1
9. Were the numerator(s) and denominato r(s) for the parameter of interest appropriate	**Yes** (**LOW RISK**): The paper presented appropriate numerator(s) AND denominator(s) for the parameter of interest (e.g. the prevalence of low back pain).	0
	**No** (**HIGH RISK**): The paper did present numerator(s) AND denominator(s) for the parameter of interest but one or more of these were inappropriate.	1
10. Summary on the overall risk of study bias	**LOW RISK**	0-3
	**MODERATE RISK**	4-6
	**HIGH RISK**	7-9

1. Hoy D, Brooks P, Woolf A, Blyth F, March L, Bain C, et al. Assessing risk of bias in prevalence studies: modification of an existing tool and evidence of interrater agreement. J Clin Epidemiol. 2012;65: 934-939.
