# Supplementary material for: JC polyomavirus (JCV, HPyV2) seropositivity prevalence in healthy subjects: Systematic review and meta-analysis
Source: PLoS One. 2026 Jan 27;21(1):e0341146. doi: 10.1371/journal.pone.0341146 (PMC12843548; doi:10.1371/journal.pone.0341146)
Supplement: S6 Table — (PDF) [file pone.0341146.s006.pdf]

S6 Table. Partial correlation between independent variables age and dependent variable prevalence controlling for region and method. a. Cells contain zero-order (Pearson) corelations.

Correlations

Control Variables			Prev	Age=0-1	Age=1-2	Age=2-14	Age=15-49	Age=50+	Region	Method
-none-[a]	Prev	Correlation	1.000	-.108	.189	-.509	.129	.309	.084	.019
		Significance (2-tailed)	.	.427	.164	<.001	.342	.020	.538	.892
		df	0	54	54	54	54	54	54	54
	Age=0-1	Correlation	-.108	1.000	-.026	-.106	-.143	-.143	.152	-.081
		Significance (2-tailed)	.427	.	.849	.438	.292	.292	.265	.554
		df	54	0	54	54	54	54	54	54
	Age=1-2	Correlation	.189	-.026	1.000	-.074	-.101	-.101	.007	.061
		Significance (2-tailed)	.164	.849	.	.587	.461	.461	.959	.656
		df	54	54	0	54	54	54	54	54
	Age=2-14	Correlation	-.509	-.106	-.074	1.000	-.410	-.410	.029	-.083
		Significance (2-tailed)	<.001	.438	.587	.	.002	.002	.833	.541
		df	54	54	54	0	54	54	54	54
	Age=15-49	Correlation	.129	-.143	-.101	-.410	1.000	-.556	-.125	-.086
		Significance (2-tailed)	.342	.292	.461	.002	.	<.001	.358	.530
		df	54	54	54	54	0	54	54	54
	Age=50+	Correlation	.309	-.143	-.101	-.410	-.556	1.000	.039	.174
		Significance (2-tailed)	.020	.292	.461	.002	<.001	.	.775	.200
		df	54	54	54	54	54	0	54	54
	Region	Correlation	.084	.152	.007	.029	-.125	.039	1.000	-.092
		Significance (2-tailed)	.538	.265	.959	.833	.358	.775	.	.499
		df	54	54	54	54	54	54	0	54
	Method	Correlation	.019	-.081	.061	-.083	-.086	.174	-.092	1.000
		Significance (2-tailed)	.892	.554	.656	.541	.530	.200	.499	.
		df	54	54	54	54	54	54	54	0
Region & Method	Prev	Correlation	1.000	-.121	.188	-.514	.145	.308		
		Significance (2-tailed)	.	.382	.174	<.001	.296	.024		
		df	0	52	52	52	52	52		
	Age=0-1	Correlation	-.121	1.000	-.023	-.118	-.135	-.142		
		Significance (2-tailed)	.382	.	.867	.397	.332	.307		

Correlations

Control Variables			Prev	Age=0-1	Age=1-2	Age=2-14	Age=15-49	Age=50+	Region	Method
		df	52	0	52	52	52	52		
	Age=1-2	Correlation	.188	-.023	1.000	-.070	-.095	-.114		
		Significance (2-tailed)	.174	.867	.	.616	.494	.412		
		df	52	52	0	52	52	52		
	Age=2-14	Correlation	-.514	-.118	-.070	1.000	-.421	-.405		
		Significance (2-tailed)	<.001	.397	.616	.	.002	.002		
		df	52	52	52	0	52	52		
	Age=15-49	Correlation	.145	-.135	-.095	-.421	1.000	-.549		
		Significance (2-tailed)	.296	.332	.494	.002	.	<.001		
		df	52	52	52	52	0	52		
	Age=50+	Correlation	.308	-.142	-.114	-.405	-.549	1.000		
		Significance (2-tailed)	.024	.307	.412	.002	<.001	.		
		df	52	52	52	52	52	0		
