# Supplementary material for: JC polyomavirus (JCV, HPyV2) seropositivity prevalence in healthy subjects: Systematic review and meta-analysis
Source: PLoS One. 2026 Jan 27;21(1):e0341146. doi: 10.1371/journal.pone.0341146 (PMC12843548; doi:10.1371/journal.pone.0341146)
Supplement: S7 Table — (PDF) [file pone.0341146.s007.pdf]

S7 Table. Independent-samples Kruskal-Wallis test output (SPPS v.29) on prevalence across age.

Hypothesis Test Summary

	Null Hypothesis	Test	Sig.[a,b]	Decision
1	The distribution of Prev is the same across categories of Age.	Independent-Samples Kruskal-Wallis Test	.006	Reject the null hypothesis.

a. The significance level is .050.
b. Asymptotic significance is displayed.

Independent-Samples Kruskal-Wallis Test Summary

Total N	56
Test Statistic	14.628[a]
Degree Of Freedom	4
Asymptotic Sig.(2-sided test)	.006

a. The test statistic is adjusted for ties.

Pairwise Comparisons of Age

Sample 1-Sample 2	Test Statistic	Std. Error	Std. Test Statistic	Sig.	Adj. Sig.[a]
2-14-0-1	3.962	12.387	.320	.749	1.000
2-14-15-49	-14.712	5.810	-2.532	.011	.113
2-14-50+	-19.212	5.810	-3.307	<.001	.009
2-14-1-2	39.462	16.924	2.332	.020	.197
0-1-15-49	-10.750	12.095	-.889	.374	1.000
0-1-50+	-15.250	12.095	-1.261	.207	1.000
0-1-1-2	-35.500	19.974	-1.777	.076	.755
15-49-50+	-4.500	5.157	-.873	.383	1.000
15-49-1-2	24.750	16.711	1.481	.139	1.000
50+-1-2	20.250	16.711	1.212	.226	1.000

Each row tests the null hypothesis that the Sample 1 and Sample 2 distributions are the same. Asymptotic significances (2-sided tests) are displayed. The significance level is .050.

a. Significance values have been adjusted by the Bonferroni correction for multiple tests.
