# Supplementary figures and images for: JC polyomavirus (JCV, HPyV2) seropositivity prevalence in healthy subjects: Systematic review and meta-analysis
Source: PLoS One. 2026 Jan 27;21(1):e0341146. doi: 10.1371/journal.pone.0341146 (PMC12843548; doi:10.1371/journal.pone.0341146)

**S1 Fig. Galbraith plot for assessing heterogeneity in the pooled meta-analysis.**

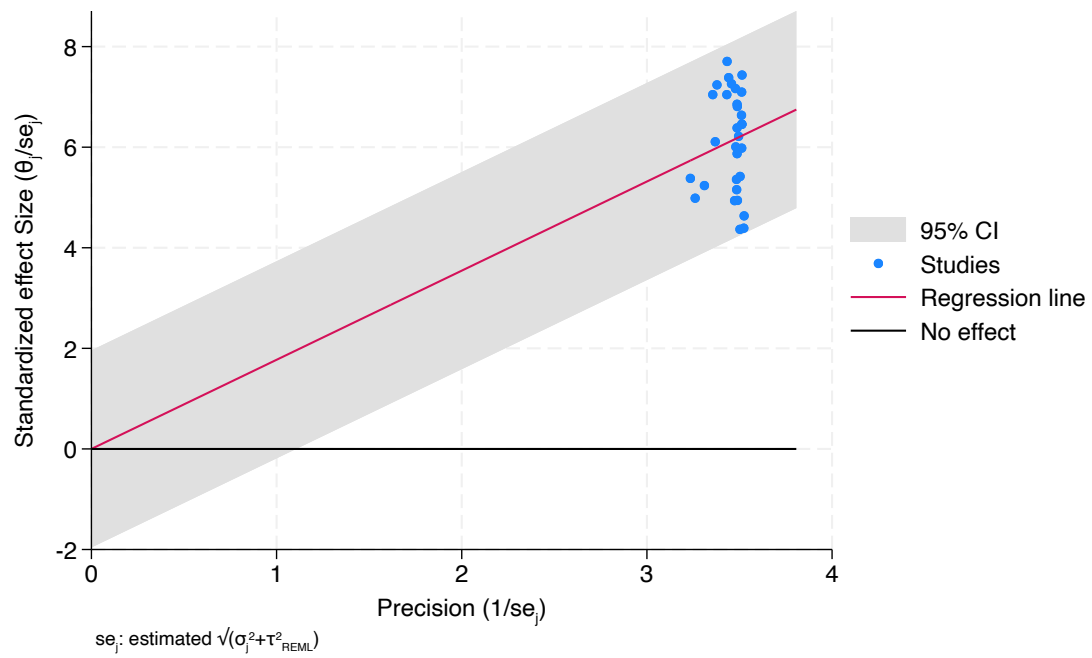

Supplement: S1 Fig — (PDF) [file pone.0341146.s009.pdf]

S3 Fig. Bubble plot with region as moderator.

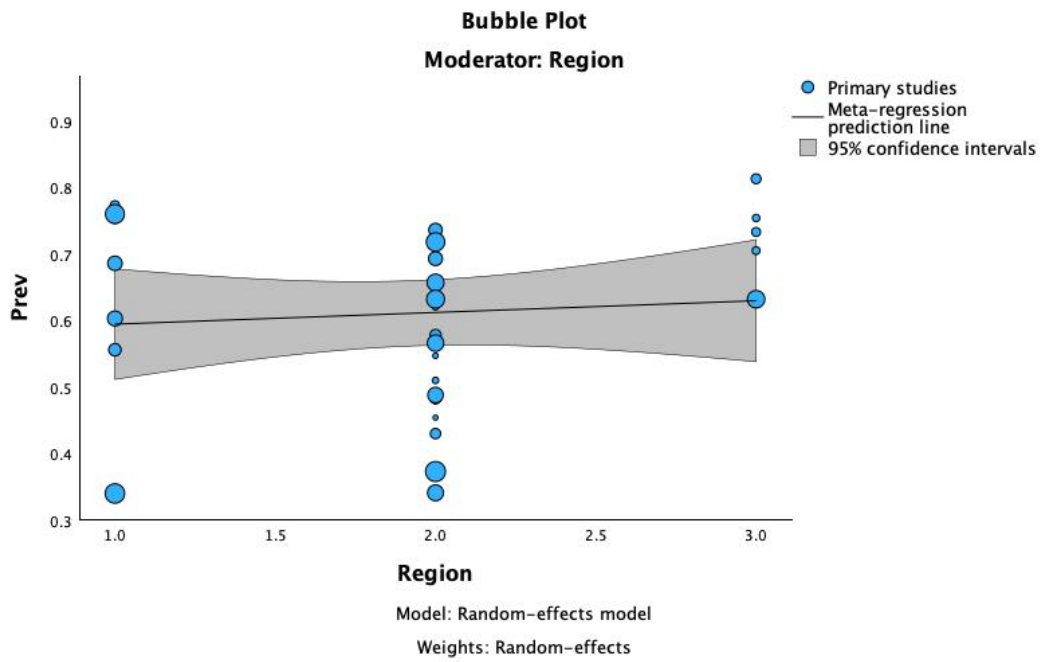

Supplement: S3 Fig — (PDF) [file pone.0341146.s011.pdf]

S4 Fig. Bubble plot with method as moderator.

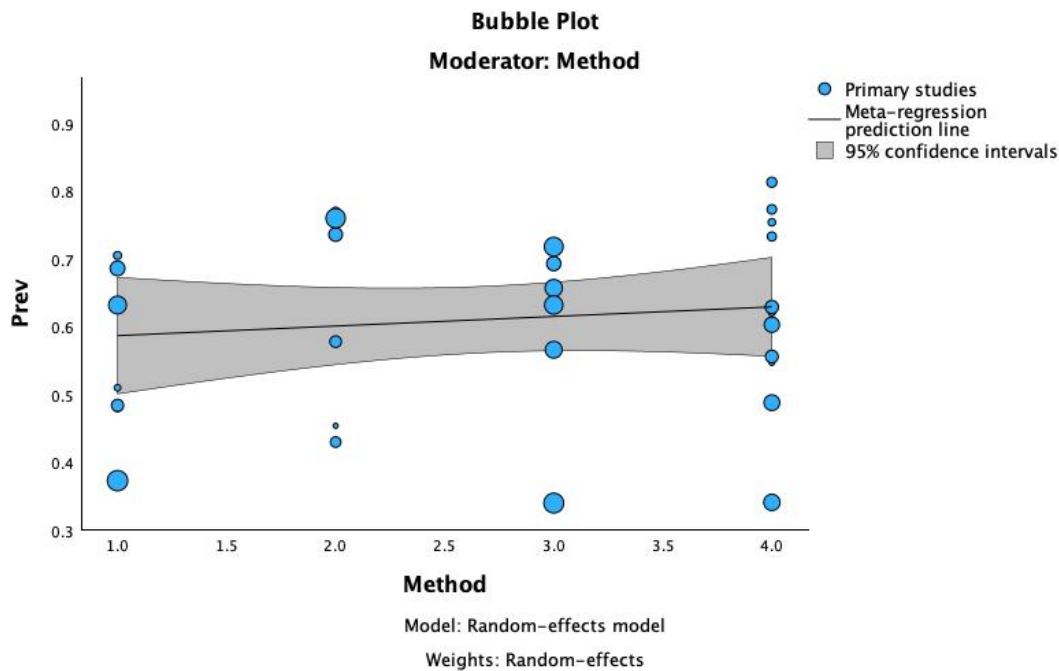

Supplement: S4 Fig — (PDF) [file pone.0341146.s012.pdf]

**S7 Fig. Funnel plot for pooled meta-analysis of JCV seropositivity for publication bias.**

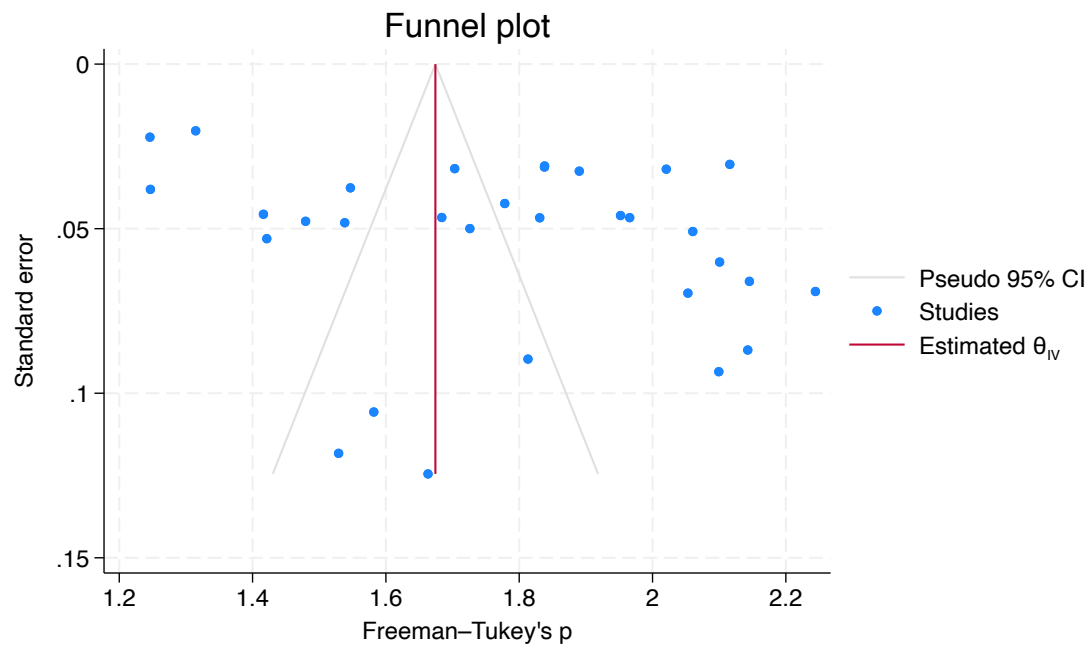

Supplement: S7 Fig — (PDF) [file pone.0341146.s015.pdf]

S9 Fig. Bubble plot with age as moderator.

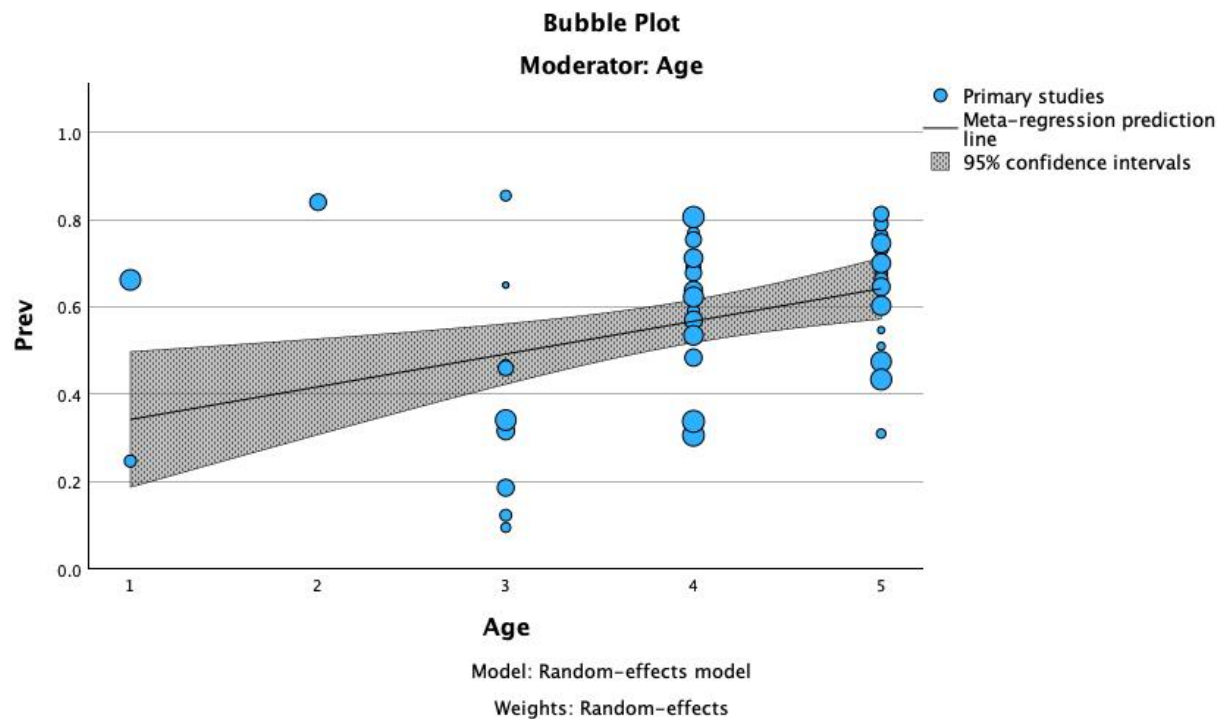

Supplement: S9 Fig — (PDF) [file pone.0341146.s017.pdf]
