# Supplementary material for: JC polyomavirus (JCV, HPyV2) seropositivity prevalence in healthy subjects: Systematic review and meta-analysis
Source: PLoS One. 2026 Jan 27;21(1):e0341146. doi: 10.1371/journal.pone.0341146 (PMC12843548; doi:10.1371/journal.pone.0341146)
Supplement: S2 Fig — (PDF) [file pone.0341146.s010.pdf]

S2 Fig. Meta-regression output from STATA 18 for pooled meta-analysis dataset.

```
Random-effects meta-regression                    Number of obs   =        31
Method: REML                                      Residual heterogeneity:
                                                              tau2 =   .08073
                                                            I2 (%) =    97.73
                                                                H2 =    44.03
                                                     R-squared (%) =     0.00
                                                  Wald chi2(2)    =      0.72
                                                  Prob > chi2     =    0.6988
```

_meta_es	Coefficient	Std. err.	z	P>|z|	[95% conf.	interval]
region	.0309113	.0781488	0.40	0.692	-.1222575	.18408
method	.0325564	.0447805	0.73	0.467	-.0552118	.1203246
_cons	1.644181	.1949249	8.43	0.000	1.262135	2.026227

```
Test of residual homogeneity: Q_res = chi2(28) = 1648.56 Prob > Q_res = 0.0000
```
