# Supplementary material for: JC polyomavirus (JCV, HPyV2) seropositivity prevalence in healthy subjects: Systematic review and meta-analysis
Source: PLoS One. 2026 Jan 27;21(1):e0341146. doi: 10.1371/journal.pone.0341146 (PMC12843548; doi:10.1371/journal.pone.0341146)

S6 Fig. Subgroup meta-analysis for JCV seroprevalence in healthy subjects by region in the pooled dataset.

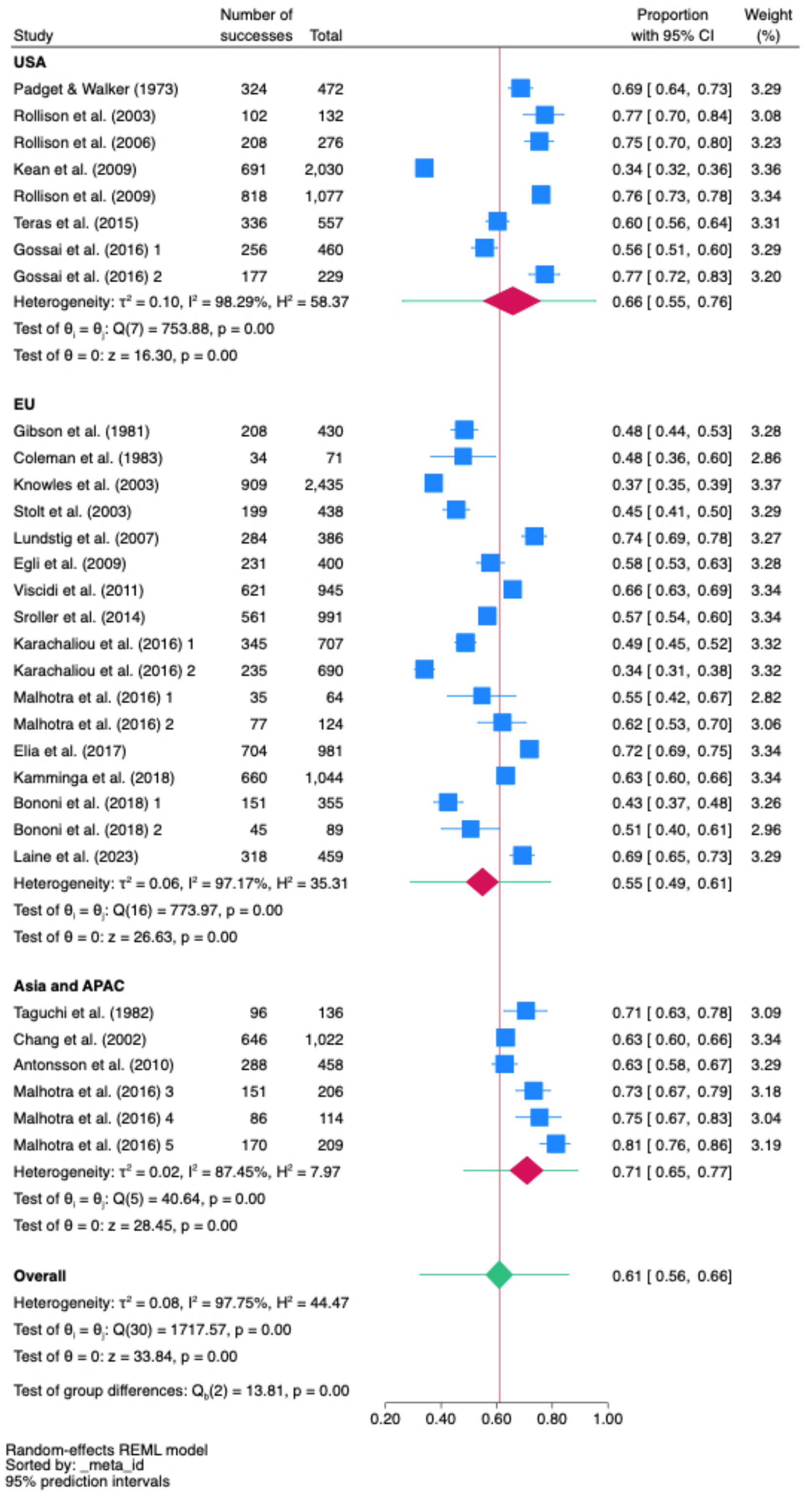

Supplement: S6 Fig — (PDF) [file pone.0341146.s014.pdf]
