# Supplementary material for: JC polyomavirus (JCV, HPyV2) seropositivity prevalence in healthy subjects: Systematic review and meta-analysis
Source: PLoS One. 2026 Jan 27;21(1):e0341146. doi: 10.1371/journal.pone.0341146 (PMC12843548; doi:10.1371/journal.pone.0341146)
Supplement: S8 Fig — (PDF) [file pone.0341146.s016.pdf]

S8 Fig. Meta-regression for the subgroup age effect on seroprevalence.

```
Random-effects meta-regression                    Number of obs  =        56
Method: REML                                      Residual heterogeneity:
                                                              tau2 =   .1426
                                                            I2 (%) =   97.70
                                                                H2 =   43.52
                                                     R-squared (%) =   12.64
                                                  Wald chi2(1)   =      8.58
                                                  Prob > chi2    =    0.0034
```

_meta_es	Coefficient	Std. err.	z	P>|z|	[95% conf.	interval]
age	.1530136	.0522422	2.93	0.003	.0506208	.2554063
_cons	1.093792	.2147976	5.09	0.000	.6727961	1.514787

Test of residual homogeneity: Q_res = chi2(54) = 2159.02 Prob > Q_res = 0.0000
