# Supplementary material for: JC polyomavirus (JCV, HPyV2) seropositivity prevalence in healthy subjects: Systematic review and meta-analysis
Source: PLoS One. 2026 Jan 27;21(1):e0341146. doi: 10.1371/journal.pone.0341146 (PMC12843548; doi:10.1371/journal.pone.0341146)

S10 Fig. Curve estimation of independent categorical variable age on dependent continuous variable prevalence.

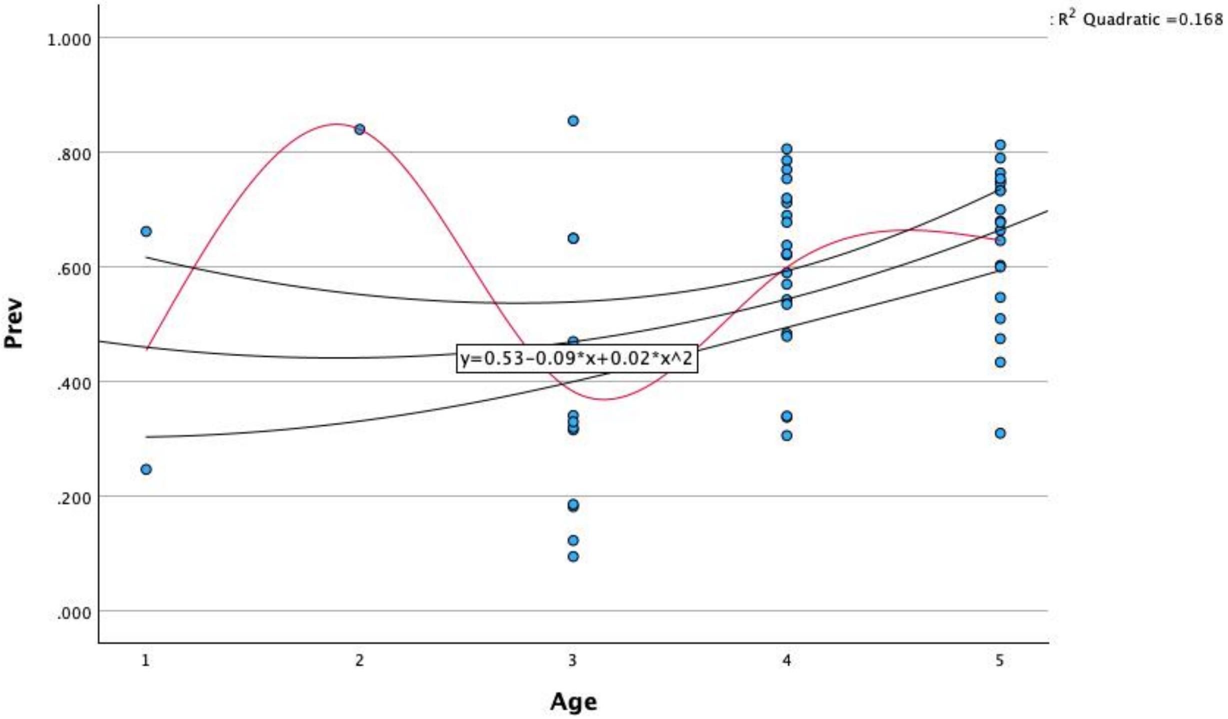

Supplement: S10 Fig — (PDF) [file pone.0341146.s018.pdf]
